# Association between Insulin-Like Growth Factor-1 and Uric Acid in Chinese Children and Adolescents with Idiopathic Short Stature: A Cross-Sectional Study

**DOI:** 10.1155/2018/4259098

**Published:** 2018-01-28

**Authors:** Panpan Wang, Baolan Ji, Qian Shao, Mei Zhang, Bo Ban

**Affiliations:** Department of Endocrinology, Affiliated Hospital of Jining Medical University, Jining, Shandong 272029, China

## Abstract

**Objective:**

The aim of this study was to examine the relationship between insulin-like growth factor-1 (IGF-1) and serum uric acid (UA) in Chinese children and adolescents with idiopathic short stature (ISS).

**Methods:**

A cross-sectional study of 91 Chinese children and adolescents with ISS was performed. Anthropometric measurements and biochemical parameters were tested. The standard deviation score of IGF-1 (IGF-1 SDS) was calculated.

**Results:**

A univariate analysis displayed a significant positive correlation between IGF-1 SDS and UA (*P* = 0.004). In multivariate piecewise linear regression, the levels of IGF-1 SDS increased with the elevation of UA when UA was between 168 *μ*mol/L and 301 *μ*mol/L (*β* 0.010, 95% CI 0.004–0.017; *P* = 0.002). The levels of IGF-1 SDS decreased with the elevation of UA when UA was either less than 168 *μ*mol/L (*β*  −0.055, 95% CI −0.081–−0.028; *P* < 0.001) or more than 301 *μ*mol/L (*β*  −0.005, 95% CI −0.013–0.002; *P* = 0.174).

**Conclusions:**

This study demonstrated a nonlinear relationship between IGF-1 and UA levels in Chinese children and adolescents with ISS. This finding suggests that either high or low levels of UA may have an adverse effect on IGF-1, whereas appropriate UA levels have a beneficial effect.

## 1. Introduction

The growth hormone-insulin-like growth factor-1 (GH-IGF-1) axis is central to the regulation of growth and development. Low levels of GH or IGF-1 can result in short stature in childhood. Usually, children with idiopathic short stature (ISS) show normal IGF-1 levels, but in some other cases IGF-1 levels can be lower (e.g., GH receptor mutations or IGF acid-labile subunit gene variants) [1, 2]. Kim et al. have demonstrated that six months of GH treatment may not only increase the growth rate and improve height standard deviation score (SDS) but also increase IGF-1 and IGF-binding protein-3 (IGFBP-3) levels in children with ISS [3].

Related studies have shown that the generation of IGF-1 was influenced by diet and nutritional status [4, 5]. Moreover, both the quantity and quality of foods intake may affect the production of IGF-1. Specifically, the levels of IGF-1 were positively correlated with the intake of red meat, fish, and oil, whereas the intake of carbohydrate foods had a negative effect on the levels of IGF-1 [6]. Additionally, starvation, semistarvation, fasting, and caloric restriction may lead to a decline in IGF-1 levels [7].

Uric acid (UA) was the final product of purine metabolism of nucleic acids. The majority of serum UA was freely filtered through the kidney glomeruli. According to preliminary estimates, approximately 90% of the filtered UA was reabsorbed by renal tubule [8], which implied that it may have a considerable physiological role in humans. Research showed that the levels of UA were influenced by a variety of internal and external factors including genetics and diet [9]. Therefore, as it was a metabolic product, the levels of UA could reflect a range of dietary intakes to some extent. In addition, studies have revealed that poor eating behaviors such as picky eating, partial eating, and even malnutrition are prevalent among children with short stature [10], which may affect the levels of UA. Previous studies on UA mainly focused on gout [11]. Recently, UA has been found to be correlated with many metabolic diseases such as obesity, metabolic syndrome, nonalcoholic fatty liver disease (NAFLD), type 2 diabetes, and cardiovascular diseases [12–16]. Regrettably, there was little related research on the relationship between IGF-1 and UA. Only Sesti et al. have demonstrated an inverse correlation between IGF-1 and UA levels in nondiabetic adults [17].

Until now, the association between IGF-1 levels and UA concentrations in children with ISS has not been studied. The aim of this study was to examine the relationship between IGF-1 and UA in Chinese children and adolescents with ISS.

## 2. Subjects and Methods

### 2.1. Study Subjects

We retrospectively reviewed the medical records of short-statured children and adolescents from the Department of Endocrinology, Affiliated Hospital of Jining Medical University, between January 2015 and December 2016. Ninety-one children and adolescents with ISS (68 males and 23 females) aged 10.1 ± 3.8 years were enrolled in this study. The subjects were selected based on the following inclusion criteria: short stature, a condition wherein the height of an individual was more than 2 SD scores (SDSs) below the corresponding mean height for a given age, sex, and population group; normal weight and height at birth; peak GH levels > 10 ng/ml in at least two GH stimulation tests. The exclusion criteria included the presence of chronic organic diseases, GH deficiency, chromosomal abnormalities, skeletal dysplasia, genetic metabolic diseases, thyroid dysfunction, and abnormal liver function, as well as the use of medication that interferes with GH secretion or function [18].

The study was approved by the Human Ethics Committee of the Affiliated Hospital of Jining Medical University (Shandong, China). All of the families of the patients were informed of the aims of the study, and informed consent was written and obtained from all patients of the subjects.

### 2.2. Anthropomorphic Measurements

Heights were measured by a specially designated individual using the same measuring instrument (produced by Nantong Best Industrial Co. Ltd., Jiangsu, China) in the morning with an allowable error range of 0.1 cm. Height SDS was calculated based on normal range of Chinese children [19]. BMI was calculated as the ratio between body weight in kilograms and height in meters squared. The stage of puberty was assessed by physical examinations according to Tanner staging [20]. The following criteria were considered as prepuberty [21, 22]: boys with a testicular volume of less than 4 mL and no pubic hair and girls with no breast development and no pubic hair.

### 2.3. Laboratory Measurements

To assess GH secretion, L-dopa (Levodopa Tablets®, He Feng, Guang Xi, China, body weight more than 30 kg, 500 mg of levodopa; less than 30 kg, 250 mg of levodopa) and insulin (Insulin Injection®, Wan Bang, Jiang Su, China, 0.1 U/kg) were administered orally or subcutaneously after overnight fasting. Blood samples were collected 0, 30, 60, 90, and 120 min later to obtain the serum GH concentration for each time point. GH was measured using a chemiluminescence method (ACCESS2, Beckman Coulter; USA) with an analytical sensitivity of 0.010 *μ*g/L. Serum IGF-1 and IGFBP-3 levels were measured by the chemiluminescence immunometric method (DPC IMMULITE 1000 analyzer, SIEMENS, Germany) with intra- and interassay CVs for IGF-1 of 3.0% and 6.2%, respectively, and intra- and interassay CVs for IGFBP-3 of 4.4% and 6.6%, respectively. Measures of liver function (including alanine aminotransferase (ALT), AST, and gamma-glutamyl transferase (GGT)), kidney function (including Cr, blood urea nitrogen (BUN), and UA), lipid profiles (including total cholesterol (TC), high-density lipoprotein cholesterol (HDL-C), LDL-C, and triglycerides (TG)), and fasting plasma glucose (FPG) were tested by a biochemical autoanalyzer (Cobas c 702, Roche; Shanghai, China). Measures of thyroid function, including Free T3 (FT3), Free T4 (FT4), thyroid-stimulating hormone (TSH), gonadotropin, cortisol rhythm, and adreno–cortico–tropic hormone (ACTH), were tested by a luminescence immunoassay system (Cobas e 602, Roche; Shanghai, China). The standard deviation score of IGF-1 (IGF-1 SDS) was calculated according to a previous study [23].

### 2.4. Statistical Analysis

**Figure 1:**
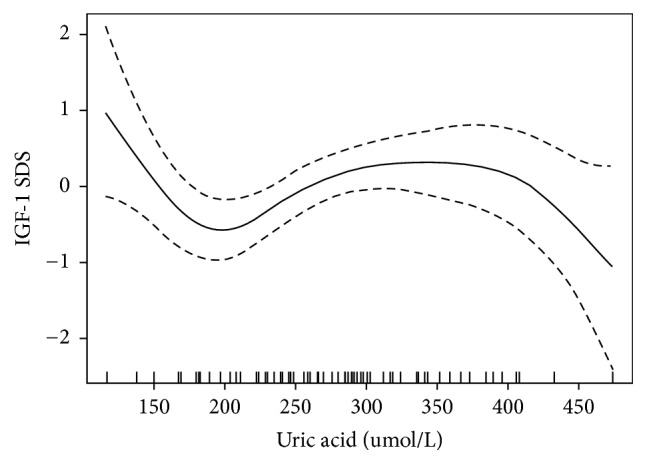
The relationship between IGF-1 SDS and UA by smooth curve fitting. Adjustment variables: body weight, pubertal stage, TG, LDL-C, Cr, and FPG. TG: triglyceride; LDL-C: low-density lipoprotein cholesterol; Cr: creatinine; FPG: fasting plasma glucose.

All of the statistical analyses were performed with R statistical software (https://www.r-project.org) and EmpowerStats (http://www.empowerstats.com, X&Y solutions, Inc. Boston MA). Normally distributed variables are expressed as the mean ± standard deviation (SD), abnormally distributed variables are shown as the median (quartile), and non-parametric tests were used. Qualitative data are expressed as the numbers and percentage (*n*, %). First, we describe the demographic characteristics and biochemical values of the subjects (Table 1). Next, a univariate analysis model (Table 2) was used to examine whether UA and other anthropometrical and biochemical variables were correlated to IGF-1 SDS. Then, we explored the relationship between IGF-1 SDS and UA by smooth curve fitting after adjustment for potential confounders (Figure 1). Finally, we further performed a multivariate piecewise linear regression model to assess the independent correlation between IGF-1 SDS and UA according to smooth curve fitting (Table 3). *P* values (two-tailed) below 0.05 were considered statistically significant.

## 3. Results

### 3.1. Clinical and Laboratory Characteristics of the Subjects

The clinical and laboratory characteristics of the subjects are shown in Table 1. A total of 91 subjects (25.3% females) with a mean age of 10.1 ± 3.8 years were included in this study. The majority of the subjects were in prepuberty (67, 73.6%). The mean levels of IGF-1 SDS and UA were −0.65 ± 1.19 and 271.39 ± 70.28 *μ*mol/L, respectively.

### 3.2. Correlations between IGF-1 SDS and Anthropometrical and Biochemical Variables

Univariate analysis was performed to analyze the relationship between IGF-1 SDS and each variable. As shown in Table 2, there was a significant positive correlation between the IGF-1 SDS and age, height, body weight, BMI, Cr, puberty stage (*P* < 0.001), and UA (*P* = 0.004), whereas a significant negative correlation was found between the IGF-1 SDS and LDL-C (*P* = 0.015) and AST (*P* = 0.001).

### 3.3. The Independent Correlation between IGF-1 SDS and UA by Multivariate Piecewise Linear Regression

Covariate screening was used to screen for possible confounders. The screening criteria included effect factors producing a >10% change when introducing covariates in the basic model or eliminating covariates in the regression model. The results revealed that body weight, pubertal stage, Cr, LDL-C, and TG met the filter criteria. In addition, smooth curve fitting was performed after adjustment for all variables, and the resultant curve exhibited a three-stage change and two breakpoints (Figure 1). Namely, there was an inverse relationship between IGF-1 SDS and UA when UA was before the first point or after the second point. However, there was a positive relationship between IGF-1 SDS and UA when UA was between the two points. Then, threshold saturation effects were analyzed based on the curve in Table 3, and the data indicated that the inflection points were 168 *μ*mol/L and 301 *μ*mol/L. Specifically, the IGF-1 SDS decreased with the elevation of UA when UA was less than 168 *μ*mol/L (*β*  −0.055, 95% CI −0.081–−0.028; *P* < 0.001) or more than 301 *μ*mol/L (*β*  −0.005, 95% CI −0.013–0.002; *P* = 0.174), whereas the IGF-1 SDS increased with the elevation of UA when UA was between 168 *μ*mol/L and 301 *μ*mol/L (*β*  0.010, 95% CI 0.004–0.017; *P* = 0.002).

## 4. Discussion

In this cross-sectional study, we demonstrated a nonlinear association between IGF-1 SDS and serum UA in Chinese children and adolescents with ISS. Namely, there was a positive relationship between IGF-1 SDS and UA when the concentration of UA was between 168 *μ*mol/L and 301 *μ*mol/L. In contrast, there was an inverse relationship between IGF-1 SDS and UA when the concentration of UA was either less than 168 *μ*mol/L or more than 301 *μ*mol/L.

UA is the end product of purine metabolism in humans. It is known that high levels of UA are associated with many metabolic diseases [11–16], whereas a recent report declared that extremely low levels of serum UA may induce endothelial dysfunction [24]. Thus, we infer that UA levels that were too high or too low may both have negative impacts on the human body. Our study indicated an inverse relationship between IGF-1 SDS and UA when the concentration of UA was less than 168 *μ*mol/L. However, there has been no research on the relationship between IGF-1 and the low levels of UA until now. Given the small sample size of this group, it is necessary to carry out related studies to explore the correlation between IGF-1 and low levels of UA and its potential mechanism. In addition, although the difference was not statistically significant, our research showed an inverse trend between IGF-1 SDS and UA when the concentration of UA was greater than 301 *μ*mol/L. This relationship was consistent with findings by Sesti et al. [17]. Furthermore, Sesti et al. noted that exposing human hepatoma cells to UA at a concentration of 300 *μ*mol/L could induce the downregulation of GH-stimulated IGF-1 expression [17]. The potential mechanism of this phenomenon was that UA may impair the ability of GH to stimulate JAK2-STAT5 signaling, which was the most important intracellular signaling pathway stimulating transcription of IGF-1 by inducing oxidative stress and post-translational changes of proteins [25]. Additionally, Guler et al. showed that IGF-1 infusion for 6 days could induce an increase in glomerular filtration rate (GFR) and a decrease in the serum UA concentration in two healthy adult subjects [26].

Notably, this study also described a positive relationship between IGF-1 SDS and UA when the concentrations of UA were between 168 *μ*mol/L and 301 *μ*mol/L, which was inconsistent with previous findings [17]. To the best of our knowledge, previous studies showed that UA levels are approximately 10-fold higher in humans than in other mammals, which means that UA levels increased in the process of human evolution [27]. This implies that UA may play an important role in the human body, rather than simply serving as a waste product of purine metabolism. Indeed, the physiological effects of UA involve many aspects, including antioxidant effects, mediation of type 2 immune responses, resistance to parasites, defense of the nervous system, and autoimmune diseases [28]. As far as our findings were concerned, although the specific mechanism remained unclear, we considered that it may differ based on the study population: previous subjects were adults [17, 26], and in vivo and in vitro studies showed that UA had detrimental effects on IGF-1, whereas our study indicated that UA was positively associated with IGF-1 in children and adolescents with ISS. There were some differences between adults and children in their eating habits and diet types. Picky eaters, partial eclipse, and even malnutrition were prevalent among children with short stature. Observational data indicated that purine content was higher in fish, seafood, meat, and the internal organs of animals [29]. UA may be used as an indicator for evaluating the intake of those foods. Indeed, Larsson et al. indicated that greater dietary intake of protein, red meat, fish, and seafood showed a significant positive association with higher IGF-1 concentrations in healthy, well-nourished men [4]. Therefore, we hypothesized that a widespread phenomenon of picky eating and partial eating may lead to inadequate intake of those foods in short children, which may affect the levels of UA and IGF-1. Adequate intake of more purine-rich foods may help increase IGF-1 levels, which was conducive to growth and development. In addition, because IGF-1 was a common indicator of the effectiveness of GH treatment, the effect of high-purine diet should be considered when using IGF-1 to assess the effectiveness of recombinant human growth hormone (rhGH) treatment.

Several potential limitations of this study should be considered. First, we failed to collect information about the diet of the subjects and did not perform a diet analysis of our study group. Therefore, we intend to design the dietary questionnaire to further analyze the effects of diet on IGF-1. Second, the levels of serum UA were influenced by diet, metabolism, and other factors. Therefore, it is necessary to measure enzymes related to UA synthesis or decomposition, such as xanthine oxidase or adenosine deaminase. In addition, the present study did not measure urinary UA levels for 24 hours, which was a stable indicator to assess the excretion of UA. Third, the number of samples in this study was relatively small, especially in the group of UA < 168 *μ*mol/L (*n* = 5). Therefore, we should further verify our results by expanding the sample size in any future studies. Finally, because of the study's cross-sectional nature, the present findings only exhibited a nonlinear relationship between IGF-1 levels and the serum UA concentration. Prospective basic and clinical studies are thus required to further confirm causality.

In conclusion, in this present study, we described a nonlinear relationship between IGF-1 and UA levels in Chinese children and adolescents with ISS after adjusting for several confounders. This finding suggests that either high or low levels of UA may have an adverse effect on IGF-1, whereas appropriate UA levels have a beneficial effect on IGF-1.

## Figures and Tables

**Table 1:** Demographic characteristics and biochemical values of the subjects.

Variables	All
Number	91
Sex (male *n*, %)	68 (74.7%)
Age (years)	10.1 ± 3.8
SBP (mmHg)	107.3 ± 12.5
DBP (mmHg)	63.9 ± 10.1
Height (cm)	126.3 ± 20.0
Body weight (kg)	27.6 ± 10.9
BMI (kg/m^2^)	16.56 ± 2.21
IGF-1 (ng/ml)	210.08 ± 148.89
IGF-1 SDS	−0.65 ± 1.19
IGFBP-3 (*μ*g/ml)	4.74 ± 1.35
Cr (*μ*mol/L)	38.70 ± 10.93
BUN (*μ*mol/L)	4.86 ± 3.13
UA (*μ*mol/L)	271.39 ± 70.28
TC (mmol/L)	3.64 ± 0.69
TG (mmol/L)	0.58 (0.48–0.86)
HDL-C (mmol/L)	1.32 ± 0.26
LDL-C (mmol/L)	1.92 ± 0.51
VLDL-C (mmol/L)	0.40 ± 0.20
ALT (U/L)	14.00 (12.00–17.30)
AST (U/L)	25.92 ± 5.34
GGT (U/L)	11.91 ± 2.55
FPG (mmol/L)	4.55 ± 0.41
Pubertal stage	
In prepuberty (*n*, %)	67 (73.6%)
In puberty (*n*, %)	24 (26.4%)

SBP: systolic blood pressure; DBP: diastolic blood pressure; BMI: body mass index; IGF-1: insulin-like growth factor-1; IGF-1 SDS: the standard deviation score of IGF-1; IGFBP-3: insulin-like growth factor-binding protein-3; Cr: creatinine; BUN: blood urea nitrogen; UA: uric acid; TC: total cholesterol; TG: triglyceride; HDL-C: high-density lipoprotein cholesterol; LDL-C: low-density lipoprotein cholesterol; VLDL-C: very low-density lipoprotein cholesterol; ALT: alanine aminotransferase; AST: aspartate transaminase; GGT: gamma-glutamyl transferase; FPG: fasting plasma glucose. Normal distribution of data was presented as mean ± standard deviation; nonnormal distribution of data was presented as median (interquartile range) and was log-transformed (base 10) before parametric tests; the categorical data was expressed by the numbers and percentage (*n*, %).

**Table 2:** Correlations between IGF-1 SDS and anthropometrical and biochemical variables.

Variables	*β*	(95% CI)	*P* value
Age (years)	0.161	(0.103, 0.219)	<0.001
Height (cm)	0.035	(0.025, 0.046)	<0.001
Body weight (kg)	0.072	(0.054, 0.090)	<0.001
BMI (kg/m^2^)	0.253	(0.150, 0.357)	<0.001
Cr (*μ*mol/L)	0.057	(0.037, 0.076)	<0.001
BUN (*μ*mol/L)	0.008	(−0.072, 0.088)	0.847
UA (*μ*mol/L)	0.005	(0.002, 0.009)	0.004
TC (mmol/L)	−0.384	(−0.790, 0.022)	0.068
TG (mmol/L)	0.660	(−0.308, 1.628)	0.186
HDL-C (mmol/L)	−0.841	(−1.856, 0.175)	0.109
LDL-C (mmol/L)	−0.704	(−1.258, −0.150)	0.015
VLDL-C (mmol/L)	1.251	(−0.096, 2.527)	0.073
ALT (U/L)	−0.022	(−0.078, 0.034)	0.435
AST (U/L)	−0.083	(−0.130, −0.036)	0.001
FPG (mmol/L)	0.145	(−0.496, 0.785)	0.659
Sex			
Male		0	
Female	−0.567	(−1.184, 0.049)	0.075
Puberty stage			
In prepuberty		0	
In puberty	1.578	(1.096, 2.016)	<0.001

BMI: body mass index; Cr: creatinine; BUN: blood urea nitrogen; UA: uric acid; TC: total cholesterol; TG: triglyceride; HDL-C: high-density lipoprotein cholesterol; LDL-C: low-density lipoprotein cholesterol; VLDL-C: very low-density lipoprotein cholesterol; ALT: alanine aminotransferase; AST: aspartate transaminase; FPG: fasting plasma glucose; *P* < 0.05 is considered to be statistically significant.

**Table 3:** The independent correlation between IGF-1 SDS and UA by multivariate piecewise linear regression.

Inflection point of uric acid (*μ*mol/L)	*β*	(95% CI)	*P* value
<168	−0.055	(−0.081, −0.028)	<0.001
168~301	0.010	(0.004, 0.017)	0.002
>301	−0.005	(−0.013, 0.002)	0.174

Adjustment variables: body weight, pubertal stage, TG, LDL-C, Cr, and FPG; TG: triglyceride; LDL-C: low-density lipoprotein cholesterol; Cr: creatinine; FPG: fasting plasma glucose; *P* < 0.05 is considered to be statistically significant.
